# A Merited Compliment

**Published:** 1858-02

**Authors:** 


### A Merited Compliment.

We take the liberty (without the knowledge of the gentleman concerned,) of announcing the fact that the Faculty of the Atlanta Medical College, as some slight evidence of their appreciation of the extraordinary energy and ability which has been manifested by Prof. John G. Westmoreland, in behalf of the Institution, have determined to place his portrait in the College edifice, to the erection of which his exertions have so largely contributed. [sen. ed.]

